# Analgesic, Anti-Inflammatory, and Antioxidant Activities of* Byrsonima duckeana* W. R. Anderson (Malpighiaceae)

**DOI:** 10.1155/2017/8367042

**Published:** 2017-03-06

**Authors:** Maria Christina dos Santos Verdam, Fernanda Guilhon-Simplicio, Kleyton Cardoso de Andrade, Karina Lorena Meira Fernandes, Tallita Marques Machado, Felipe Moura Araújo da Silva, Mayane Pereira de Souza, Hector Henrique Ferreira Koolen, Cristiane da Silva Paula, Beatriz Cristina Konopatzki Hirota, Vinícius Bednarczuk de Oliveira, Cristina Mayumi Sasaki Miyazaki, Milena Kalegari, Marilis Dallarmi Miguel, Patricia Maria Stuelp-Campelo, Obdulio Gomes Miguel

**Affiliations:** ^1^Department of Pharmacy, Federal University of Paraná, Curitiba, PR, Brazil; ^2^Faculty of Pharmacy, Federal University of Rio de Janeiro, Rio de Janeiro, RJ, Brazil; ^3^Faculty of Pharmaceutical Sciences, Federal University of Amazonas, Manaus, AM, Brazil; ^4^Center of Biological and Health Sciences, Pontifical Catholic University of Paraná, Curitiba, PR, Brazil; ^5^Department of Chemistry, Federal University of Amazonas, Manaus, AM, Brazil; ^6^DeMpSter Mass Spectrometry Group, Amazonas State University, Manaus, AM, Brazil

## Abstract

*Background*.* Byrsonima *is a promising neotropical genus, rich in flavonoids and triterpenes, with several proven pharmacological properties. Nevertheless,* Byrsonima duckeana *W. R. Anderson is an Amazonian species almost not studied.* Objective*. To assess the antioxidant, anti-inflammatory, and analgesic activities of* Byrsonima duckeana *leaves.* Materials and Methods*. We analyzed an ethanol extract and its fractions for polyphenol content and UHPLC-MS/MS, phosphomolybdenum, DPPH, TBARS antioxidant tests, formalin-induced pain, carrageenan-induced peritonitis, acetic acid-induced abdominal writhings, and hot plate assays.* Results*. All the samples showed high polyphenol content and antioxidant capacity in the phosphomolybdenum, DPPH, and TBARS tests. We identified ethyl gallate, quinic acid, gallic acid, catechin, epicatechin, quercetrin, and quercetin in the samples.* B. duckeana* was able to reduce leukocyte migration in the carrageenan-induced peritonitis by 43% and the licking time in the formalin test by 57%. In the acetic acid-induced writhing test, the chloroform (FCL) and ethyl acetate (FEA) fractions were the most active samples. FEA was selected for the hot plate test, where all the dosages tested (5, 50, and 200 mg·kg^−1^) showed significant analgesic activity.* Conclusion*.* B. duckeana *has interesting analgesic and antioxidant activities, due to its high phenolic content, especially phenolic acids.

## 1. Introduction

Inflammation is a response of immune system that activates many enzymatic and cellular processes to protect the body from all kinds of trauma [1]. This process is associated with pain [2], which is caused by the stimulation of afferent nervous fibers by inflammatory chemical mediators, whose primary function is to protect the organism [3]. Despite the importance of the inflammatory process in providing protection, pain is one of the most frequent factors that motivates individuals to seek medical help and is a cause of low productivity in the workplace, absence due to sickness, and early retirement [4].

Beside inflammatory pain, other usual kinds of pain are nociceptive and neuropathic. Although they have separate mechanisms, both nociceptive and neuropathic types of pain share physiological aspects with the inflammatory pain. The first is mostly caused by the sensitization of primary nociceptive neurons through direct action of inflammatory mediators due to tissue damage [5]. Nociceptive pain is characterized by sensitization of the nerve fibers by receptors and ionic channels that are sensitive to heat, cold, and protons. Neuropathic pain is caused by a lesion or disease affecting the somatosensory nervous system [6].

The* Byrsonima *genus, a member of the Malpighiaceae family, has more than 100 described species [7], distributed through neotropical regions [8]. Species of this genus have attracted the interest of researchers by their antimicrobial activity against gram-positive, gram-negative, and enteric bacteria, along with mycobacteria, protozoa, and fungi. Other biological activities, such as spasmogenic, immunostimulatory, anti-inflammatory, antihemorrhagic, antihyperglycemic, antihyperlipidemic, antiulcer, antidiarrheal, and antioxidant activities, were also investigated in different species [9].

*Byrsonima duckeana* W.R. Anderson is a tree found in the Brazilian Amazon rainforest, about which we previously reported promising cytotoxicity on colon cancer cells [10]. However, its pharmacological and chemical potential are still underexplored, since we already observed* in vivo* activities of other Amazonian* Byrsonima* species,* Byrsonima japurensis*, that has promising anti-inflammatory, analgesic, and antioxidant activities, corroborating its popular use [11]. Thereby, this study evaluated the chemical composition and the antioxidant, anti-inflammatory, and analgesic activities of the ethanolic extract and its fractions from the leaves of* B. duckeana*, in order to better evaluate its* in vivo* pharmacological potential.

## 2. Materials and Methods

### 2.1. Plant Material

The leaves from a flowering plant of* Byrsonima duckeana* were collected in November 10, 2010, in the Adolpho Ducke Reserve, Manaus, Amazonas State, Brazil, from a specimen previously catalogued during the “Flora da Reserva Ducke” project [12], when a voucher (#179696) was deposited in the Herbarium of the National Institute of Research in the Amazon (INPA). The Institute Chico Mendes for Biodiversity Conservation provided authorization (#41553-1) from the Brazilian Ministry of Environment for the plant collection.

### 2.2. Preparation and Fractionation of Crude Extract

Leaves from* B. duckeana* were dried at 40°C in a convection oven. The plant material was crushed and submitted to extraction in a Soxhlet apparatus. The material was extracted with ethanol until exhaustion, yielding a crude extract named EEB, used in the tests. An aliquot of EBB was suspended in ethanol and partitioned with hexane, chloroform, and ethyl acetate, three times each, affording fractions FHX, FCL, and FEA, respectively.

### 2.3. Determination of Total Phenolics

The extract and its fractions were submitted to the Folin-Ciocalteu colorimetric method for determination of their phenolic content by a previously described methodology [13]. The analysis was performed in triplicate, and the results were expressed in mg of gallic acid equivalents (GAE.100 g^−1^).

### 2.4. DPPH Radical Scavenging

Aliquots of the samples (2.5 mL) were incubated in the absence of light and at room temperature with a 2,2-diphenyl-1-picrylhydrazyl (DPPH) radical solution (300 *μ*M, 40 mL) for 30 minutes. Thus, absorbance was recorded at 518 nm in triplicate to check the bleaching of the mixture, indicating the radical scavenging. Ascorbic acid and rutin were used as positive controls, and methanol was used as the negative control [14].

### 2.5. Phosphomolybdenum Spectrophotometric Method

Aliquots of the plant samples (0.3 mL, 200 *μ*g·mL^−1^) in methanol were added to 3 mL of the phosphomolybdenum reagent and were allocated at 95°C during 90 minutes, and then the absorbance was measured at 695 nm in triplicate. The results were expressed as percentages of antioxidant activity in comparison with ascorbic acid and rutin [15].

### 2.6. Thiobarbituric Acid Reactive Substances (TBARS)

Aliquots of each sample (100 *μ*L = 100 ppm) were suspended in ethanol. Further, egg yolk solution (500 *μ*L), 2,2′-azobis(2-amidinopropane)-dihydrochloride (ABAP, 50 *μ*L, 0.035%), acetic acid (1.5 mL, 20% v/v), and thiobarbituric acid (TBA, 1.5 mL, 0.4% m/v) were added, and the reaction occurred for 60 minutes at 95°C. Positive control was butylated hydroxytoluene (BHT). The final reaction mixture was then extracted with* n*-butanol and centrifuged for 3 minutes. The absorbance values of the supernatants were measured at 532 nm to detect the formation of malondialdehyde (MDA) indicating the decomposition of polyunsaturated fatty acid compounds [16]. The analysis was performed in quintuplicate.

### 2.7. Phytochemical Analysis

We used a HPLC–MS system, consisting of a Shimadzu SPD-M10 apparatus coupled to a Bruker Amazon ion-trap mass spectrometer. Electrospray ionization (ESI) was used to explore the phenolic composition of* B. duckeana* leaf extract and fractions. Chromatographic separation was performed on a Phenomenex Luna C18 column (5 *μ*m, 250 × 4.6 mm) using a binary mobile phase. Solvent A was water and solvent B was methanol. A gradient elution at 30°C was performed as follows: 0–3 min, 5% (v/v) B; 3–40 min, 5–70% (v/v) B; 40–44 min, 70–100% (v/v) B; and 44–60 min, 100% (v/v) B, at a flow rate of 0.4 mL·min^−1^. The autosampler temperature was held at 20°C and the injection volume was 10 *μ*L. The ESI source operated in negative mode for the identification of the polyphenols [17]. Ionization parameters were as follows: VCap, 3500 V; nozzle voltage, 0 V; fragmentor, 100 V; skimmer, 65 V; gas temperature, 280°C; gas flow, 14 L min^−1^; and nebulizer, 45 psi. The MS and MS/MS spectra were acquired at the* m/z* range from 50 to 1000. Tentative identifications were performed by manual interpretation of MS/MS spectral data with those previously reported [17–19].

### 2.8. Animals

We performed experiments with male Swiss mice (25–35 g) for the crude EEB and with both sexes, in groups equally balanced, for the comparison between EEB and its respective fractions. The animals were housed in polypropylene cages at 25 ± 2°C with a 12 h light-dark cycle and acclimatized in the experimental environment for at least 24 h before the tests. Water and a balanced diet were continually provided ad libitum, but food was withdrawn 12 h before the tests. All experiments were approved by the Ethics Committee for Animal Research of the Federal University of Amazonas (UFAM, protocol number 048/2011) and Pontifical Catholic University of Paraná (PUC-PR, protocol number 675, second version).

### 2.9. Acute Toxicity

A maximum dosage of 2000 mg·kg^−1^ of the samples studied in this work was dissolved in normal saline solution and administered orally to different groups with each consisting of eight mice. Abnormal morphological and behavioral signs of toxicity were evaluated during the first 4 h after administration of* B. duckeana* samples [20] and at 24 h intervals, for 14 days [21]. For the negative control group only saline solution was administered.

### 2.10. Formalin-Induced Pain Test

Four groups of eight mice each received 20 *μ*L of a formalin solution (2.5%, v/v) by intraplantar injections into the ventral surface of the right hind paw. One hour before injections, two groups received EEB at 500 and 1000 mg·kg^−1^, orally. The total time spent by each mouse on licking its paw was recorded with a hand chronometer. The animals were observed for 30 minutes, according to each phase of the test: neurogenic (0–5 minutes) and inflammatory phases (15–30 minutes), respectively. At the end of the test, the animals were euthanized and the inflamed paws were sectioned and weighed to measure the formed edema. Morphine at 3 mg·kg^−1^ and the saline solution were used as positive and negative controls, respectively [22, 23].

### 2.11. Carrageenan-Induced Peritonitis Test

Three groups of eight mice received injections of 250 *μ*L of carrageenan in normal saline solution (1%, v/v) into the peritoneum. Two of the groups previously received EEB at 500 and 1000 mg·kg^−1^ by the oral route. Four hours after the carrageenan injection, the mice were euthanized by an intraperitoneal injection of a mixture of xylazine (350 mg·mL^−1^) and ketamine (40 mg·mL^−1^). After the euthanasia of animals, the peritoneal cavity was washed with 2 mL of phosphate buffer saline (PBS); then, the collected peritoneal fluid was centrifuged at 3000 rpm for 10 minutes. The supernatant was used to determine the total amount of proteins in the exudate, by measuring the absorbance at 280 nm. The cell pellets were diluted in a mixture sodium phosphate buffer and Turk solution (1 : 10, v/v) and used for the total leukocyte counting [24].

### 2.12. Acetic Acid-Induced Writhing Test

Six groups of six mice each were used for this experiment. Four different groups received fraction samples, being 100 mg·kg^−1^ of each fraction to one group (EEB, FHX, FCL, and FEA) by oral administration. The positive control group received indomethacin at a dosage of 10 mg·kg^−1^, and the negative control group received the normal saline solution. After 60 minutes, all mice received an intraperitoneal injection of an acetic acid solution (1%, v/v in saline solution). The abdominal writhings were counted cumulatively during 20 minutes after the injection, and the total cumulative number in this period was taken as an indicator of the nociceptive response [25].

### 2.13. Hot Plate Test

Different groups of the six mice received dosages of 5, 50, and 200 mg·kg^−1^ of FEA orally. Other two groups received 10 mg·kg^−1^ of indomethacin (positive control) and normal saline solution (negative control). The animals were placed individually on a digital hot plate (Insight EFF-361) previously heated to 52 ± 2°C, and the typical behavior was observed at time 0 (to obtain a baseline) and at 30, 60, 90, and 120 minutes after the fraction administration [21].

### 2.14. Statistical Analysis

The results were presented as mean  ± standard deviation when applicable. Since no difference was observed in the response of female or male animals, both sexes were considered in the statistical analysis between the groups. The results of each group in the* in vivo* analysis were compared by the Tukey test, considering (*p* < 0.05) values as statistically significant. The median inhibitory concentrations (IC_50_) of antioxidant activity were calculated by using linear regression. The statistical analysis was carried out with GraphPad Prism 5.0 Software (GraphPad Software, Inc. La Jolla, USA).

## 3. Results

### 3.1. Total Phenolics and Antioxidant Capacity

Within the analyzed samples in this study, FEA presented the highest amount of total polyphenols. In the DPPH test, all results presented statistically significant differences (*p* < 0.05) and FCL and FEA showed the best antioxidant capacity of the tested samples. In the phosphomolybdenum test, the same fractions were also the most active samples, but FEA and rutin showed no statistical difference between the obtained results, indicating that they are equivalent. In the evaluation of inhibition of the lipid peroxidation (TBARS), FEA presented the highest inhibitory potential of all tested samples (Table 1).

### 3.2. Phytochemical Analysis

**Figure 1:**
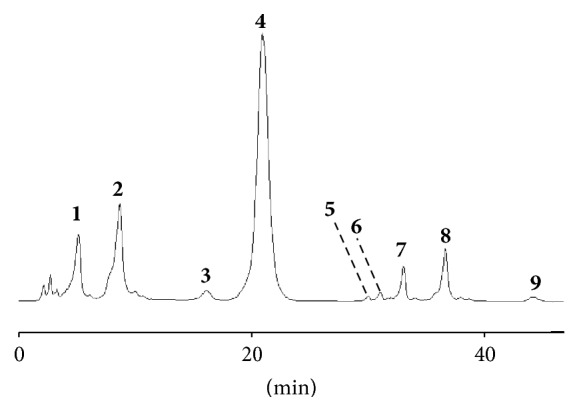
UHPLC-MS/MS chromatogram of the ethanolic extract of* B. duckeana* (EEB) showing the following compounds: quinic acid (**1**), gallic acid (**2**), unknown (**3**), ethyl gallate (**4**), catechin (**5**), epicatechin (**6**), quercetrin (**7**), quercetin (**8**), and unknown (**9**).

The EEB extract was analyzed by LC-MS/MS and direct infusion electrospray ionization tandem mass spectrometry (DI-ESI-MS/MS) (Figure 1). Fractions FCL and FEA were analyzed only by DI-ESI-MS/MS due to the low complexity observed for their corresponding extracts.

The deprotonated ions [M–H]^−^ and their corresponding fragments from CID experiments were compared to fragmentation routes previously described [18]. For the analysis of EEB, the peak eluting at 5.71 min displayed the deprotonated molecule at* m/z* 191, which fragments to the ion of* m/z* 173 (−18 Da; neutral loss of water) and* m/z* 127 (−46 Da; neutral loss of formic acid), allowing to identify it as quinic acid. Further product ions at* m/z* 111, 93, and 85 reinforced this structure proposal [25]. Peak** 2** (*R*_*t*_ 9.53 min) was tentatively identified as the gallic acid based on its deprotonated molecule at* m/z* 169 and the characteristic carbon dioxide loss (−44 Da), giving the fragment [M–CO_2_–H]^−^ at* m/z* 125 [26]. The main peak (**4**), eluting at 20.37 min, was tentatively assigned as ethyl gallate based on its typical fragmentation ions (*m/z* 197 → 169 and* m/z* 169 → 125), related to those observed for the gallic acid [17].

Peaks** 5** and** 6** eluted at 29.84 and 30.63 min, respectively. Both peaks displayed the same MS/MS spectra, where the deprotonated ion (*m/z* 289) displayed CID fragments consistent with those reported for the catechins. Based on the product ion and on the elution order previously reported, peaks** 5** and** 6** were assigned as (+)-catechin and (−)-epicatechin, respectively [19]. The compound that eluted at 33.09 min (peak** 7**) displayed a deprotonated ion at* m/z* 447, with a glucose loss as first fragmentation (−162 Da), yielding the product ion at* m/z* 301. This and the other corresponding fragments are in accordance with the tentative identification of quercetrin for this peak elution. Its corresponding aglycone was observed eluting at 36.15 min (peak** 8**) [27]. The less intense peaks** 3** and** 9** displayed uncommon fragmentation behaviors, being both assigned as unknown compounds.

**Figure 2:**
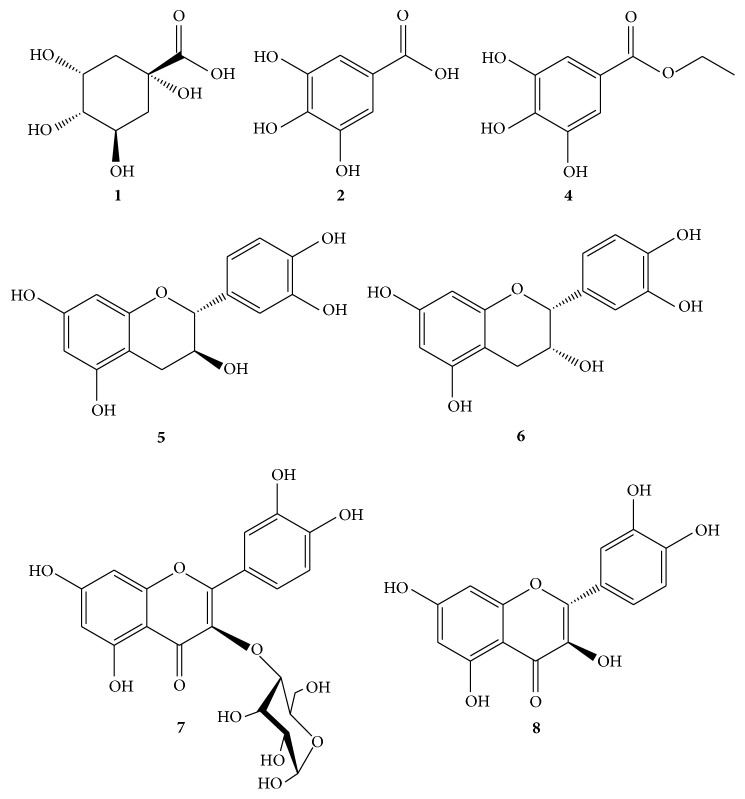
Polyphenols identified in* B. duckeana*.

Phenolic acids and esters and some flavonoids were identified in the two most promising fractions FCL and FEA. The main compounds of FCL were quinic acid, gallic acid, and ethyl gallate. In minor proportions, catechin, quercetin and quercetrin were observed. Ethyl gallate was the main compound of FEA (Figure 2).

### 3.3. Acute Toxicity

The toxicity of the EEB sample was analyzed. No deaths in the groups of animals that received EEB at the maximum dosage were observed and no macroscopic signs of harmful effects were observed in any of the animals; thus, the LD_50_ was not calculated.

### 3.4. Formalin-Induced Pain Test

**Figure 3:**
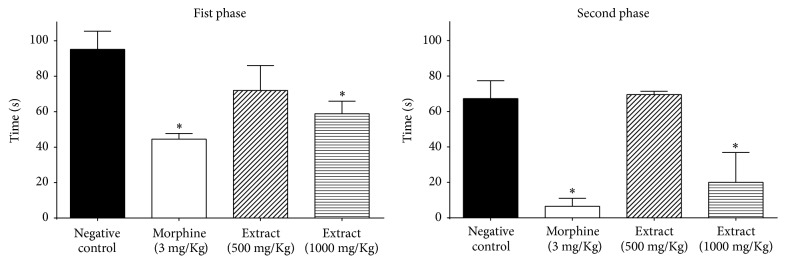
Results of the preliminary screening of analgesic potential of* Byrsonima duckeana*. ^*∗*^*p* < 0.05 versus negative control.

In this assay, EEB inhibited the inflammatory phase (15–30 minutes) and the neurogenic one (0–5 minutes) in the dosage of 1000 mg·kg^−1^ (Figure 3).

### 3.5. Carrageenan-Induced Peritonitis Test

The number of leukocytes that migrated to the site of the inflammation local was 45.25 ± 9.84% lower than in the negative controls, at the dosage of 500 mg kg^−1^ of EEB, and 51.95 ± 5.94% lower at the dose of 1000 mg·kg^−1^, without statistical differences between them. The protein exudation was lowered by 12.79 ± 2.61% and 45.38 ± 8.55% for both dosages, respectively (*p* < 0.05). This previous analysis showed that the activity of EEB reduced the leucocyte migration, suggesting that extract may control the symptoms of the acute inflammation; however, only the dosage of 1000 mg·kg^−1^ interfered significantly with protein exudation.

### 3.6. Acetic Acid-Induced Writhing Test

**Figure 4:**
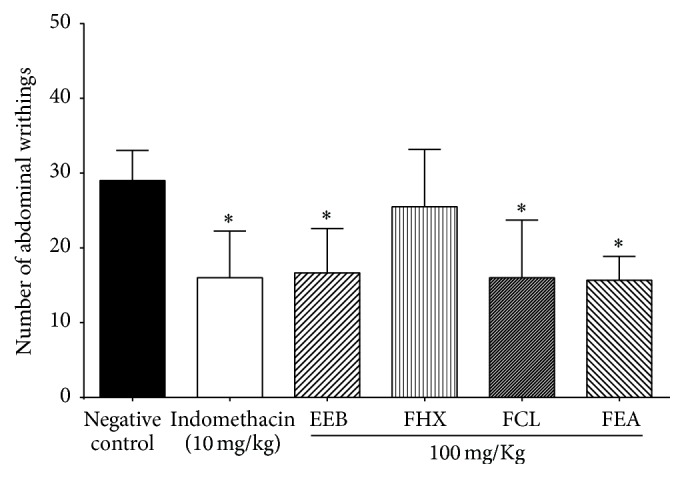
Effect of crude extract and fractions of* Byrsonima duckeana *on acetic acid-induced abdominal writhing. EEB: crude ethanolic extract. FEA: ethyl acetate fraction. FCL: chloroform fraction. FHX: hexane fraction. ^*∗*^*p* < 0.05 versus negative control.

To perform the screening of samples in the writhing test, a dosage of 100 mg·kg^−1^, commonly used in* in vivo* pharmacological assays of crude plant extracts and prepurified fractions were selected. The analysis of variance between the mean counts of abdominal writhings in each group showed that EEB, FCL, and FEA present statistically significant analgesic activity (*p* < 0.05) in comparison with the negative control (normal saline) (Figure 4).

### 3.7. Hot Plate Test

**Figure 5:**
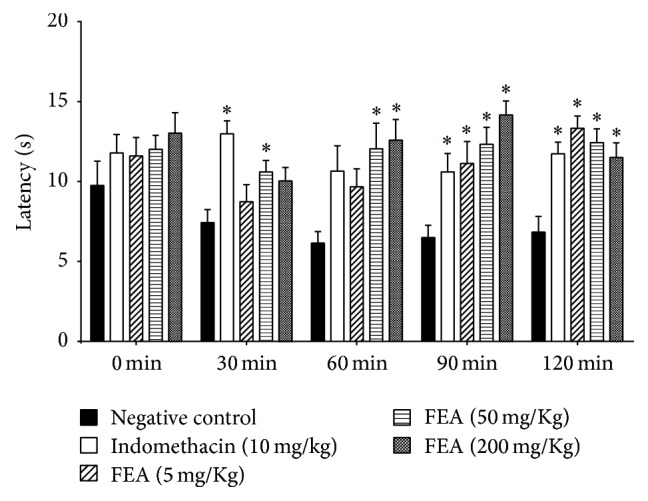
Effect of ethyl acetate fraction (FEA) of* Byrsonima duckeana *on the hot plate test. Maximal activity of all the dosages was observed at 90 minutes. ^*∗*^*p* < 0.05 versus negative control.

For the hot plate test, only FEA was administrated to the mice (5, 50, and 200 mg·kg^−1^). FEA at a dosage of 200 mg·kg^−1^ showed significant activity when compared with the negative control for all analyzed periods. At 90 and 120 minutes, all tested dosages showed analgesic activity. None of them showed differences in comparison with positive control (indomethacin 10 mg·kg^−1^). FEA showed antinociceptive activity equivalent to that of the standard drug, even at the lowest dosage tested, 5 mg·kg^−1^, half of the dose positive control, revealing FEA, as a promising source of substances with analgesic activity (Figure 5).

## 4. Discussion

In this study, which aimed to investigate the pharmacological potentials of* B. duckeana*, an evaluation of its anti-inflammatory, analgesic, and antioxidant abilities was carried out, considering the empirical medicinal use of plants of this genus.

Since polyphenols are considered the most active antioxidant metabolites from plants, the relationship between polyphenol concentration and antioxidant activity has been the focus of many studies [28, 29]. The mass spectrometry analysis highlighted that the phenolic constitution of FEA is mainly composed of traces of gallic acid in association with high amounts of its corresponding ethyl ester. These results are in agreement with the observed anti-inflammatory actions of FEA, once antioxidant compounds like phenol acids and esters can inhibit the formation of peroxynitrite (ONOO^−^) from the combination of superoxide radical (O_2_^−•^) and nitric oxide (NO) released during the inflammatory response (membrane damage, lipid peroxidation) [11]. These reactive oxygen species (ROS) have been implicated in the pathogenesis of various diseases, including rheumatoid arthritis, asthma, atherosclerosis, and Alzheimer's disease and, furthermore, critically involved in various pain conditions, including neuropathic and inflammatory pains [30]. These ROS are also involved in the promotion of the induction of hyperalgesia by prostaglandin E2 [31].

In the acute toxicity analysis, because of no toxicity at the doses tested, we assume that EEB only causes toxicity at higher dosages than those tested, indicating that the crude ethanolic extract is safe for administration through the oral route. These results reinforce previous observations that* Byrsonima* spp are safe for* in vivo* studies [9]. Based on this result, dosages of 500 and 1000 mg·kg^−1^ were selected for the preliminary investigation of the anti-inflammatory/antihyperalgesic screening using the carrageenan-induced peritonitis model and the formalin test.

The inflammatory response in a carrageenan-induced pleurisy culminates in exudation and leukocyte infiltration, which release chemical mediators responsible for the cardinal signals of inflammation, namely, edema, pain, heat, redness, and loss of function [32]. It is clear that protein exudation is more closely related to edema than to other events [33]. These chemical mediators released by the leucocytes and other recruited cells are responsible, for example, for the hypersensitization of nerve fibers, which cause inflammatory pain or hyperalgesia [34]. This previous analysis showed that the activity of EEB reduced leucocyte migration, suggesting that the extract may control the symptoms of acute inflammation; however, only the dosage of 1000 mg·kg^−1^ interfered significantly with protein exudation.

In order to evaluate the action of EEB in an acute inflammation model, we deployed the formalin test, which is a satisfactory model for evaluating the anti-inflammatory/antinociceptive activity of plant extracts and isolated molecules. In the first phase of the test, intraplantar injections of formalin produce neurogenic pain, which combines a peripheral input and a spinal cord sensitization [32]. This phase is associated with an acute pain that starts immediately after the injection and involves nociceptors. The second phase is an inflammatory pain response inhibited by traditional analgesic and anti-inflammatory drugs [35].

Based on such observations, EEB presented antihyperalgesic capacity, but without statistical difference in comparison with the positive control (morphine 3 mg·kg^−1^). On the other hand, it was observed that none of the EEB amounts significantly reduced the edema in this test, as determined by weighting the paws, which is in agreement with the results observed in carrageenan-induced peritonitis model. In order to better investigate the analgesic action of* B. duckeana*, EEB and its respective fractions were evaluated by an abdominal writhing test.

Abdominal writhings in mice are characterized by abdominal contractions and rotations, followed by the extension of one or both hind legs. Acetic acid is a convenient stimulus for screening tests as it produces a response whose intensity depends on the interaction of many events that determine the nociception and is sensitive to analgesic substances of central and/or peripheral action, with different mechanisms of action [21].

In the writhing test, no differences were found in comparison with the positive control and, thus, it is possible to assume that the activities were equivalent. The results of this test showed that compounds with medium polarity (FCL and FEA) are more directly related to the analgesic activity of EEB. Thus, FEA was selected for the hot plate test in order to investigate if its analgesic activity is due to central action of its compounds, as previously suggested in formalin test.

Hot plate test is a central model of nociception, which is based on a high-intensity phasic stimulus [36]. Two behavioral responses are observed in this test, paw licking and jumping, being both considered supraspinally integrated responses and the timing of the latency to the onset of this response after administration is an indicative of the analgesic activity [34].

Thus, it is possible to infer that the antioxidant potential of* B. duckeana* may be related to the anti-inflammatory activity, and in particular, to the antihyperalgesic activity observed. The hot plate test, in which* B. duckeana* displayed positive results, is a classic model for the evaluation of antinociceptive activity. In this case, the mechanism of this activity requires further investigation, although there is evidence that ROS are also involved in the mechanism of the nociceptive pain [37].

## 5. Conclusion

This investigation displayed the pharmacological potential of* B. duckeana*, one of the few Amazonian native species of* Byrsonima* scientifically studied. The results indicate that this species has a promising analgesic activity, appearing to have action in both hyperalgesic and nociceptive pain, which requires further investigation in order to better explain their mechanism of action. Nevertheless, the observed effects may be related to polyphenols, abundant in the extract and respective fractions. The ethyl acetate fraction displayed strong antioxidant capacity, including free radical scavenging and inhibition of lipid peroxidation, which may lead to the improvement of pain relief. Furthermore, the chemical investigation using the ethyl acetate fraction showed that ethyl gallate is a major constituent, which can explain strong antioxidant activity observed. Furthermore, the species have also shown an interesting phenolic composition, including quercitrin, which has been identified in* Byrsonima* specie for first time.

## Figures and Tables

**Table 1:** Phenol content and antioxidant activity of *Byrsonima duckeana*.

Samples	Total phenols^*∗*^ (EqGA/g)	DPPH (IC_50_ in *μ* ± *σ* *μ*g/mL)	PES (% of AA)	TBARS (% inhibition of lipid peroxidation)
Ethanolic extract	466.60	14.88 ± 0.04	38.15 ± 0.00	76.95 ± 0.01
Hexane fraction	108.84	38.51 ± 0.09	24.20 ± 0.00	70.57 ± 0.02
Chloroform fraction	582.46	6.24 ± 0.02	60.29 ± 0.01	74.79 ± 0.02
Ethyl acetate fraction	743.74	8.22 ± 0.04	42.46 ± 0.00	88.69 ± 0.01
Ascorbic acid	NT	4.60 ± 0.14	100	NT
Rutin	NT	6.01 ± 0.01	40.94 ± 0.01	NT
BHT	NT	NT	NT	21.54 ± 0.02

NT: not tested; ^*∗*^data shown in gallic acid equivalents.
